# Severe acute respiratory syndrome coronavirus-2-associated cholangiopathies

**DOI:** 10.1097/MOG.0000000000000808

**Published:** 2021-12-22

**Authors:** Alessandra Bartoli, Carmela Cursaro, Pietro Andreone

**Affiliations:** a Division of Internal Medicine, Department of Medical and Surgical Sciences, Maternal-Infantile and Adult; b Postgraduate School of Allergy and Clinical Immunology, University of Modena and Reggio Emilia, Modena, Italy; c Chief of Postgraduate school of Allergy and clinical immunology University of Modena and Reggio Emilia, Modena, Italy

**Keywords:** cholangiopathy, coronavirus disease 2019, primary biliary cholangitis, secondary sclerosing cholangitis

## Abstract

SARS-CoV2 is a β-coronavirus, isolated for the first time in Wuhan in December 2019. Bilateral interstitial pneumonia is the hallmark of this disease. Liver is the second viral target for frequency and AST and ALT elevation is a common finding. From February 2020, two different cholangiopathies have been reported in COVID-19 patients. The aim of this article is to review the cases so far described in order to share information and awareness about these new clinical entities.

**Recent findings:**

SARS-CoV2 seems to trigger autoimmunity and two cases of primary biliary cholangitis (PBC) have been developed after viral infection while more than 30 patients have showed a rapidly progressing cholangiopathy with features of secondary sclerosing cholangitis (SSC). For what concerns SSC pathogenesis, a theory combining multiple hits is the most recognized.

**Summary:**

Two different cholangiopathies have been reported in patients after severe-COVID-19. Attention should be paid to the development of cholestasis in ICU setting but above all after discharge and liver function tests should be, therefore, periodically performed. No treatment strategies are available and liver transplantation remains the last option in individuals with liver failure because of SSC. Other efforts are necessary to better understand the pathogenesis and to expand therapeutic options.

## INTRODUCTION

Severe acute respiratory syndrome coronavirus-2 (SARS-CoV-2) is a β coronavirus isolated for the first time in Wuhan, China in December 2019. Coronavirus disease 2019 (COVID-19) is characterized by fever, cough, anosmia, ageusia, sometimes diarrhoea, culminating in bilateral interstitial pneumonia followed, in some cases, by an abnormous activation of the immune system which, if not controlled, determines severe complications, such as acute respiratory distress syndrome (ARDS), heart failure, acute renal insufficiency, liver failure and death [1]. Liver is the second viral target for frequency and mild–moderate alteration in liver enzymes is often seen in hospitalized patients [2]. From SARS-CoV2 pandemic, two different diseases, both affecting the biliary tract, have been reported. The first one shows an autoimmune origin and has the features of primary biliary cholangitis (PBC) whereas the second displays the characteristics of a secondary sclerosing cholangitis and its pathogenesis is uncertain [3^▪^,^4^,^5^]. In this review, we focused our attention on patients’ common features, treatment received, imaging and histology findings in order to establish a disease profile and clarify the debated pathogenesis options. 

**Box 1:**
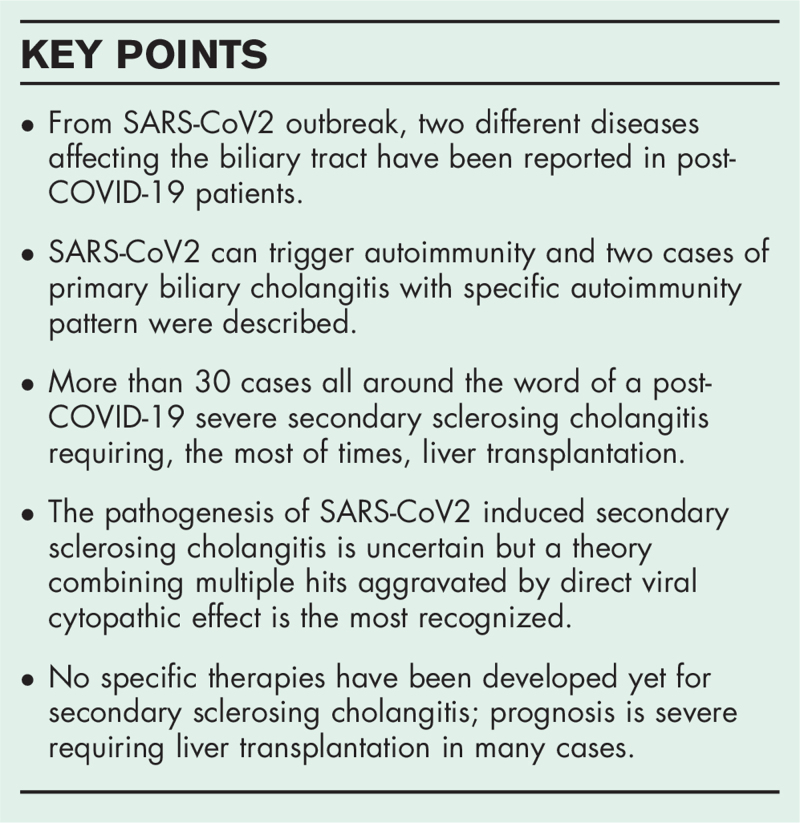
no caption available

## LIVER DISEASE IN CORONAVIRUS DISEASE 2019

During COVID-19 hospitalization, a variable percentage of patients, ranging from 14 to 76%, develops transaminase alterations with a greater elevation of aspartate aminotransferase (AST) than alanine aminotransferase (ALT) [2]. These alterations are generally mild and can often be seen at the beginning of the hospital stay, frequently referred to ischaemic hepatitis phenomena [4–6]. In the American gastroenterological association (AGA) study, liver test abnormalities were observed more frequently in United States patients [7]. Increase of cholestasis indexes are rarer but can reach the 12% of patients, with total bilirubin levels higher than three times the upper limit normal (ULN) [2,4]. In some series of patients, severe and chronic cholangiopathy accounted for less than 1% of hospitalized subjects, and in some others for 4% of mechanically ventilated ones [8^▪^,^9^]. It must be also underlined that an increase in AST, ALT and total bilirubin is a risk factor for poor outcome in severe COVID-19 [10]. SARS-CoV2 could reach the liver via blood or gut through the bile enterohepatic circulation; gut cells in fact are provided of a large number of angiotensin-converting enzyme-2 (ACE-2) receptors, fundamental for viral entry [11]. Hepatocytes poorly express ACE-2 receptors but present large amounts of transmembrane protease serine-2 (TMPRSS2) whereas cholangiocytes and endothelial cells highly express ACE-2 receptors, becoming a possible SARS-CoV2 target [12,13]. SARS-CoV-2 virions were detected in the endothelial cells of portal veins whereas viral detection in hepatobiliary tissue differs from case to case [14^▪^].

Viral inclusions and SARS-CoV2 RNA were isolated in hepatocytes and cholangiocytes in autoptic studies whereas in other histology and pathology reports they were not observed [4,15^▪^,16^▪^,^17^].

The pathogenesis of liver involvement in COVID-19 is unclear [18]. Interleukin 6 (IL-6) is thought to be the key point of endotheliopathy and coagulopathy, and some researchers have studied its influence in the development of COVID-19-related liver damage [19^▪▪^]. Through the trans-signalling pathway, IL-6 can interact with cells that are not representing its classical targets and among them, liver sinusoidal endothelial cells (LSECs) can be found. LSECs, stimulated by IL-6, produce higher amounts of procoagulant factors (von Willebrand factor and factor VIII), pro-inflammatory mediators (IL-6, chemokine C-X-C ligand 1-2) and adhesion molecules (intercellular adhesion molecule 1, P and E-selectin), which mediate platelet aggregation and attachment and neutrophils recruitment [19^▪▪^]. COVID-19-related intrahepatic microangiopathy seems to hit three different vascular compartments with hepatic artery endothelial swelling, portal vein phlebitis, and sinusoid obstruction syndrome [20]. Sinusoidal thrombosis is a sort of hallmark in severe COVID-19 liver histology, suggesting a possible direct viral action in the pathogenesis of the disease [5].

## POST CORONAVIRUS DISEASE 2019 CHOLANGIOPATHIES

At the moment, two distinct diseases have been reported in literature. The occurrence of an autoimmune disease with the characteristics of primary biliary cholangitis and a large duct cholangiopathy resembling secondary sclerosing cholangitis [3^▪^,^4^,^5^].

### Severe acute respiratory syndrome coronavirus-2-induced primary biliary cholangitis

SARS-CoV2 demonstrated to trigger autoimmunity, in fact, two cases of PBC developing after viral infection were described [3^▪^,^21^]. In the first case, a 47 years old woman who developed a Guillain–Barré syndrome during ICU stay, showed, after discharge, a severe elevation of gamma glutamyl-transpeptidase (GGT) and alkaline phosphatase (ALP) without jaundice. At liver ultrasound and MRI no abnormalities were noticed but antinuclear antibodies (ANA) and antimitochondrial antibodies (AMA) were highly positive and the liver biopsy was compatible with the diagnosis of PBC [3^▪^].

The other case is that of a 57 years old man who, 1 month after a mild COVID-19 not requiring hospital admission, developed nausea, abdominal pain and vomiting accompanied by itch with AST, ALT and GGT elevation in concert with hypergammaglobulinemia. AMA, antismooth-muscle antibodies and antidouble stranded DNA antibodies were positive and an autoimmune hepatitis overlapping with PBC was diagnosed [21].

### Severe acute respiratory syndrome coronavirus-2-induced secondary sclerosing cholangitis

#### General features

From February 2020, other cases of a post-COVID-19 cholangiopathy, different from that previously reported, have been described. Up to now, more than 30 patients all around the world have developed a rapidly progressive cholangiopathy requiring, in some cases, orthotopic liver transplantation (OLT) [4,5,8^▪^,^9^,16^▪^,^22^–^26^]. Subjects’ common features were a severe COVID-19 bilateral interstitial pneumonia, ICU admission, acute hypoxemic respiratory insufficiency needing mechanical ventilation with positive-end expiratory pressure (PEEP) up to 10–15 mBar, prone-positioning, the administration of a mix of different drugs and in some cases, circulation support through pressor agents, extracorporeal membrane oxygenation (ECMO) and dialysis. Patients were for the major part men with an age range from 29 to 77 years, no preexisting liver disease, or at most liver steatosis, suffering from obesity, hypertension and diabetes. These subjects showed a moderate increase in AST and ALT, early, at hospital admission, which tended to normalize during hospital stay. During ICU stay or later, after ICU discharge, they presented an increase in ALP, GGT and total bilirubin often accompanied by jaundice. These alterations tended to augment despite cardiovascular and pulmonary recovery [4,5,8^▪^,^9^,16^▪^,^22^–^26^]. ALP and GGT often tended to reach values higher than 10 times ULN. Hepatotropic viruses and liver autoimmunity were negative [24,25].

Table 1 lists the main demographical and clinical features of described cases with peak values of transaminases and cholestasis indexes.

**Table 1:** Patients’ general features with demographic data and information regarding treatments, mechanical ventilation duration, complications occurred during the acute phase of COVID-19 with cholestatic enzymes peak values and timing

Reference	Numberr of subjects	Sex	Age (years)	Underlying conditions	Body mass index (BMI, Kg/m^2^)	Drugs administered during hospital stay	Mechanical ventilation (MV) duration (days)	Pressor agents	Sedation	Complications	GGT, ALP (U/L) and total bilirubin (TB, mg/dl) peak
Mallet V, *et al.* Intravenous ketamine and progressive cholangiopathy in COVID-19 patients. *J Hepatol.* 2021 May;74(5):1243-1244	5	M 60%F 40%	Range 35–65;median 59	3/5 hypertension,2/5 diabetes,1/5 kidney transplantation,1/5 HBV infection in treatment with entecavir,1/5 resolved hepatitis B	Range 21–33; median 28	Not specified	Median 40	5/5norepinephrine for 10 (2–15) days	5/5 ketamine	5/5 acute kidney failure;3/5 dialysis	AST and ALT elevation early after admission. GGT 30.7× ULN, ALP 9.2× ULN, TB 18× ULN
Roth NC, *et al.* Post-COVID-19 Cholangiopathy: A Novel Entity. *Am J Gastroenterol*. 2021 May 1;116(5):1077-1082	3	M 67%F 23%	Range 25–40;median 34	1/3 diabetes	Not reported	3/3 hydroxichloroquine, 3/3 azithromycine, 1/3 ivermectin, 2/3 corticosteroids, 2/3 tocilizumab, 2/3 anakinra, 1/3 convalescent plasma, 1/3 remdesivir, 3/3 antibiotics	MV and tracheostomy until day 63 (2/3) and day 112 (1/3)	3/3, drug not specified	Not reported	3/3 acute kidney failure; 2/3 dialysis; 1/3 ECMO; 2/3 biventricular systolic heart failure, 2/3 secondary infection, 1/3 cardiac arrest	AST and ALT elevation early after admission. GGT not reported,ALP 16× ULN at day 139 (pt 1), 103 (pt 2), 172 (pt 3);TB 20× ULN at day 181 (pt 1), 105 (pt 2) and 182 (pt 3)
*Faruqui S, et al.* Cholangiopathy After Severe COVID-19: Clinical Features and Prognostic Implications. *Am J Gastroenterol.* 2021 Jul 1;116(7):1414-1425	12	M 92%F 8%	Range 38–73; median 58	9/12 hypertension, 5/12 diabetes, 5/12 obesity, 1/12 hepatic steatosis without cirrhosis,3/12 cardiovascular disease, 6/12 hyperlipemia	Not reported	7/12 hydroxychloroquine, 10/12 azithromycin, 2/12 tocilizumab, 2/12 remdesivir, 8/12 anticoagulants, 4/12 heparin prophylaxis, 1/12 sarilumab, 1/12 clazakizumab	Median 60, range 13-138	10/12, drug not specified	Not reported	12/12 sepsis, 8/12 thrombosis, 3/12 ECMO, 8/12 pronation	AST and ALT elevation early after admission. GGT not reported,ALP 10× ULN andTB 35× ULN.Days from COVID-19 diagnosis to cholangiopathy discovery: 117.7 +/- 18
Durazo FA, Nicholas AA, *et al.* Post-Covid-19 Cholangiopathy-A New Indication for Liver Transplantation: A Case Report. Transplant Proc. 2021 May;53(4):1132-1137	1	M	47	Severe obesity, obstructive sleep apnoea, hypertension, hyperlipidaemia; no history of liver disease	51	Hydroxychloroquine, azithromycin and high dose vitamin C	29	Not reported	Not reported	Acute kidney injury, ECMO and dialysis (permanent)	AST and ALT elevation early after admission. GGT not reported,ALP 1644 U/L, TB 19 mg/dl.Chemistry peak was registered at day 58 from hospital admission
Tafreshi S, *et al.* A case of secondary sclerosing cholangitis due to COVID-19. Clin Imaging. 2021 Jul 27;80 : 239–242	1	M	38	None	Not reported	Hydroxychloroquine, azithromycin, tocilizumab	Not reported	Not reported	Not reported	Acute kidney failure, ECMO, dialysis and embolic stroke	AST and ALT elevation early after admission. GGT not reported,ALP 3665 U/lTB 9.8 mg/dlPeak registered at a few months from hospital admission
Lee A, *et al.* Liver transplantation for post-COVID-19 sclerosing cholangitis. BMJ Case Rep. 2021 Aug 26;14(8):e244168.	1	M	64	Hypertension, hyperlipaemia, diabetes and radical prostatectomy for cancer	29.8	Hydroxychloroquine, azithromycin, tocilizumab, convalescent plasma, fluconazole and anticoagulants	14	Not reported	Not reported	Bilateral limbs deep venous thrombosis and consequent inferior vena cava filter placement; line-related candidemia	AST and ALT elevation early after admission. ALP and TB peak at day 24 after admission; jaundice at day 51 after admission
Klindt C, *et al.* Secondary sclerosing cholangitis as a complication of severe COVID-19: A case report and review of the literature. Clin Case Rep. 2021 May 24;9(5):e04068	1	M	47	None	Not reported	Lopinavir/ritonavir, remdesivir, piperacillin/tazobactam, clarithromycin and meropenem	Not reported	2 days, drug not specified	Not reported	Not reported	AST and ALT elevation early after admission. GGT, ALP and TB gradually increased from admission with GGT (28.6× ULN) and ALP (9.91× ULN) peak at day 51 and TB (21.63× ULN) peak at day 144
Rojas M, *et al.* Cholangiopathy as part of post-COVID syndrome. J Transl Autoimmun. 2021;4 : 100116	1	F	29	Obesity	Not reported	Antibiotics, colchicine, enoxaparin, aspirin, dexamethasone and furosemide	Tracheostomy due the long lasting intubation.MV duration not specified	Not reported	Not reported	Acute kidney injury, troponin elevation and sepsis	After 2 months from admission, jaundice and ALT, AST, ALP and TB elevation

ALT, alanine aminotransferase; ALP, alkaline phosphatase; AST, aspartate aminotransferase; COVID-19, coronavirus disease 19; ECMO, extracorporeal membrane oxygenation; GGT, gamma glutamyl transpeptidase; Nr, number; pts, patients; TB, total bilirubin; ULN, upper limit normal.

MRI and magnetic resonance cholangiopancreatography (MRCP) common findings were mild dilatations and strictures of intrahepatic bile ducts with ‘beaded’ appearance and biliary casts [4,8^▪^,16^▪^,^23^,^24^]. In many cases, alterations of the common bile duct, in form of dilatation were observed, not always accompanied by strictures [4,8^▪^,^24^].

Some patients performed endoscopic retrograde cholangiopancreatography (ERCP) with papilla sphincterotomy and common bile duct stenting or balloon dilatation, without benefits even in case of biliary sludge and stone elimination [8^▪^,^22^,^25^]. The vast majority of subjects, as last step, underwent liver biopsy and the main common findings consisted in portal and periportal fibrosis, bridging fibrosis, inflammatory infiltrate (leucocytes and rare times plasma cells) in portal tracts, degenerative cholangiocytes injury with cytoplasm vacuolization, regenerative changes and terminal bile ducts and marginal ductules cholangiocytes necrosis. Cytokeratin 7 immunostain positivity was observed in some biopsy samples, indicating ductular reaction. Other findings consisted in acute and/or chronic large duct obstruction and vascular damage, in particular, hepatic artery endothelial swelling, hepatic veins endophlebitis and in a case, focal features of sinusoidal obstruction syndrome [4,5,8^▪^,16^▪^,^22^,^24^].

The disease has thereby the features of a secondary sclerosing cholangitis.

MRI, MRCP, ERCP and biopsy findings of the reported cases are listed in Table 2.

**Table 2:** Diagnostic examinations performed in patients with post-coronavirus disease 2019 cholangiopathy

Reference	CT/MRI/MRCP	ERCP	Biopsy	Prognosis
Mallet V, *et al.* Intravenous ketamine and progressive cholangiopathy in COVID-19 patients. J Hepatol. 2021 May;74(5):1243-1244	Strictures and dilatation of intrahepatic bile ducts, biliary casts and peribiliary cysts	One of three filling defects in common bile duct and depletion of intrahepatic bile ducts. One biliary cast removed	Two of five: severe biliary cirrhosis and fibrosis; 4/5: cholangio- proliferation, biliary plugs and portal inflammation with leucocyte infiltrate.	Two of five died for decompensated cirrhosis and biliary sepsis.Three of five survived: 1/5 pruritus and 2/5 recurrent biliary sepsis.
Roth NC, *et al.* Post-COVID-19 Cholangiopathy: A Novel Entity. *Am J Gastroenterol* 2021 May 1;116(5):1077-1082.	Two of three hepatomegaly, 1/3 extrahepatic bile ducts dilatation and 1/3 intrahepatic bile ducts strictures and dilatations with beaded aspect or solely dilatation	Two of three: sphincterotomy with stones and sludge expulsion but no clinical benefits	Two of three mild and moderate bile ducts paucity; 3/3 moderate ductular reaction; 3/3 cholangiocytes swelling and regenerative changes with portal tract inflammation; 3/3 hepatic artery endothelial swelling, hepatic veins endophlebitis and 1/3 focal features of sinusoidal obstruction syndrome. 1/3 bridging portal fibrosis and 3/3 periportal fibrosis	Three of three still alive, 2/3 discharged home, 1/3 still hospitalized. Clinical conditions not specified
Faruqui S, *et al.* Cholangiopathy After Severe COVID-19: Clinical Features and Prognostic Implications. *Am J Gastroenterol* 2021 Jul 1;116(7):1414-1425	Eleven of twelve beaded images of intrahepatic bile ducts, 7/12 bile ducts thickening and hyperenhancement, 10/12 peribiliary diffusion high signal	Three of twelve papilla sphinterotomy and stenting of the common bile duct (CBD), balloon dilatation of strictures in left and right hepatic ducts without clinical benefits. Multiple strictures in intrahepatic bile ducts observed	Performed in four of twelve pts. Acute or chronic large bile ducts obstruction, mild fibrosis of some portal tracts, Keratin 7 immunostain positivity	Four of twelve died for complications consequent to sclerosing cholangiopathy. 1/12 rejected from transplantation program because of old age and multiorgan failure (last TB 35 mg/dl), 2/12 listed for transplantation, 1/12 LT from a living donor, 4/12 not listed for LT and take UDCA. UDCA slightly improved some lab tests (AST and ALT) but GGT and ALP remained elevated
Durazo FA, Nicholas AA, *et al.* Post-Covid-19 Cholangiopathy-A New Indication for Liver Transplantation: A Case Report. *Transplant Proc* 2021 May;53(4):1132-1137	Mild intrahepatic bile ducts dilatation with focal strictures and beaded aspect, no dilatation of CBD	Sphinterotomy and a small stone extraction without clinical benefits. Intrahepatic ducts with short segmental strictures and dilatations	Inflammatory mononuclear infiltrates of bile ducts walls with increased collagen deposition, liver abscesses and bile lakes. Endothelial cell swelling, lumen obliteration of arterial vessels and obliterative portal venopathy	At day 108 from hospital admission, orthotopic liver transplantation (OLT). Seven months after LT, normal liver function, normal ALP and TB.
Tafreshi S, *et al.* A case of secondary sclerosing cholangitis due to COVID-19. *Clin Imaging* 2021 Jul 27;80 : 239-242	Mild dilatation of intrahepatic bile ducts with beaded aspect, dilatation of CBD and periportal oedema	Attenuated and sinuous intrahepatic bile ducts with normal extrahepatic ducts	Cholangiocytes injury, ductular proliferation, canalicular cholestasis, a bile lake and focal bridging fibrosis	Under evaluation for LT
Lee A, *et al.* Liver transplantation for post-COVID-19 sclerosing cholangitis *BMJ Case Rep* 2021 Aug 26;14(8):e244168.	CBD dilatation, mild intrahepatic bile ducts dilatation and diffuse biliary hamartomas	Ductopenia of the left and right ducts with beaded appearance; filling defect suggesting biliary casts	Explant pathology: bridging fibrosis, severe bile duct injury, ductular reaction and leucocytes and plasma cells infiltrate	LT after 259 days from COVID-19 hospital admission. Eight months after LT, the liver function and cholestasis indexes are in range
Klindt C, *et al.* Secondary sclerosing cholangitis as a complication of severe COVID-19: A case report and review of the literature. *Clin Case Rep* 2021 May 24;9(5):e04068	Alterations of medium and small intrahepatic bile ducts	Not performed	Enlarged portal tracts with phlogistic infiltrate, ductular reaction with degenerative alterations of bile duct epithelium; focal biliary metaplasia of periportal hepatocytes. A few bile infarcts and perivenular canalicular cholestasis	LT
Rojas M, *et al.* Cholangiopathy as part of post-COVID syndrome. *J Transl Autoimmun* 2021;4 : 100116	Only a cystic lesion in liver segment VII	Negative for any alteration	Low periportal phlogistic infiltrate without necrosis but with a severe obstructive cholestatic pattern	UDCA and cholestyramine were administered and just a slightly improvement was observed. Even if the autoimmunity profile panel was negative, the authors think these tests should be repeated without the hyper bilirubin confounding factor. This case is different from the other ones described above.

ALT, alanine aminotransferase; ALP, alkaline phosphatase; AST, aspartate aminotransferase; CBD, common bile duct; CT, computed tomography; ERCP, endoscopic retrograde cholangiopancreatography; GGT, gamma glutamyl transpeptidase; LT, liver transplantation; MV, mechanical ventilation; MRCP, magnetic resonance cholangiopancreatography; OLT, orthotopic liver transplantation; pts, patients; TB, total bilirubin; UDCA, ursodeoxycholic acid; ULN, upper limit normal.

#### Pathogenesis

Some authors related this disease to the use of ketamine as general anaesthetic for sedation during mechanical ventilation, in COVID ICUs [23]. Ketamine has been used during the pandemic as off-label second line agent and postketamine cholangiopathy is a known rare side effect [27,28]. Mallet *et al.* observed five patients developing a post-COVID-19 cholangiopathy after mechanical ventilation and sedation with ketamine. Liver injury seemed to be dose-dependent and progressive [23]. Bütikofer *et al.* compared 34 COVID-19-intubated patients with 34 influenza intubated patients. Four of 34 in the first group versus none from the second developed a severe cholangiopathy. Ketamine was used for sedation in the entire first group and for none from the second [26]. Ketamine in fact is metabolized in the liver through nitrogen demetilation to norketamine, a water-insoluble intermediate, which can precipitate in biliary ducts determining ductal structural alterations and consequently, cholestasis [23]. The drug may, therefore, represent a second hit on a liver already compromised by other factors [29].

Other authors have indeed associated post-COVID-19 cholangiopathy to secondary sclerosing cholangitis in critically ill patients (SSC-CIP) [4,5,8^▪^,^9^,16^▪^,^22^,^24^–^26^]. This is a rare complication affecting patients who have been long time hospitalized in the ICU for different causes, needing for mechanical ventilation [30]. SSC-CIP patients have neither history of prior liver disease nor cholangiopathy and even if the pathogenesis is uncertain, hypoxemia of the hepatosplanchnic district may represent the leading cause, in particular, due to systemic hypotension for prolonged time [31]. Mechanical ventilation with PEEP higher than 10 cm H_2_O can determine microcirculatory ischemia of the splanchnic plexus [32]. The prone position for long time during mechanical ventillation and the administration of pressor agents as norepinephrine, epinephrine and dobutamine can also decrease the hepatosplanchnic blood-flow [33]. SSC-CIP mortality reaches high rates and is up to 50% during ICU stay [34]. In a study, 60% of affected patients survived; 20% needed OLT and the remaining developed a biliary cirrhosis [35]. Precipitating factors are concomitant renal failure, a rapid deterioration of liver function with liver insufficiency and cirrhosis and a high model for end-stage liver disease (MELD) score [34]. Without OLT, the average survival is 12–44 months [36]. Even if similarities among the two clinical entities are evident, histology seems to differ; biopsies performed in post-COVID-19 cholangiopathy showed diffuse degenerative cholangiocyte lesions with extreme cytoplasm vacuolization and regenerative change, never mentioned in SCC-CIP [5]. For what concerns post-COVID-19 secondary sclerosing cholangitis, a theory combining multiple hits or the combination of SSC-CIP with direct viral damage are the most accepted [4,5,8^▪^,^22^].

The hypoxic factor, as described above, can also be applied to post-COVID-19 cholangiopathy [37]. Moreover, as SARS-CoV2 severe infection is associated with blood hypercoagulability and thrombosis, hypoxia of the hepatosplanchnic district could be worsened [38]. Cholangiocytes are more sensitive than hepatocytes to ischaemia because of their specific vascularization. In case of local ischaemia, biliary epithelium develops necrosis and sloughing with consequent bile casts production and bile ducts structural alterations [31].

Another debated co-factor consists in drug-induced live injury (DILI) [39]. COVID-19 severe patients are, in fact, administered with different drugs mix. Hydroxychloroquine, azithromycin, remdesivir, lopinavir/ritonavir, tocilizumab, corticosteroids, antifungal drugs and antibiotics were the medications most frequently given [4,5,8^▪^,16^▪^,^22^,^24^,^25^]. DILI is a diffuse phenomenon that can be caused by direct cytotoxicity or idiosyncratic reactions and sclerosing cholangitis-like diseases have been reported in literature [39]. None of the above cited drugs is known to cause cholangiopathies [40].

A third hit may be represented by the hyperactivation of the immune system leading to the ‘cytokine storm’. The most important mediators of this complication are pro-inflammatory cytokines, such as IL-1, IL-6 and TNF-α, with IL-6 being considered as key point in endotheliopathy and inflammation in COVID-19 disease. Inflammation-mediated SSC has been reported in some autoinflammatory diseases [18,41].

The last causative factor could be represented by SARS-CoV2 direct cytopathic effect as cholangiocytes are known to express large amounts of ACE-2 receptors. The alteration of normal functioning and homeostasis of cholangiocytes because of multiple factors (ischaemia but also hyperinflammatory environment and direct viral effect) may lead to the subversion of self-protective cell mechanisms and toxic bile damage. In order to protect themselves from bile acid aggression, cholangiocytes secrete bicarbonates whereas hepatocytes secrete phospholipids. An alteration of this secretory equilibrium may cause biliary epithelium damage and consequent biliary ducts sclerosis and SSC development [31].

#### Treatment and prognosis

In the reported cases, ursodeoxycholic acid and cholestyramine were of modest benefit on pruritus and did not arrest disease progression.

Prognosis remained severe, requiring liver transplantation in many patients [24,26]. In a case series of 12 patients, 4 of 12 died, 5 of 12 were listed for OLT, 1 of 12 was rejected from OLT list because of ineligibility. Six of 12 patients repeated MRI after 6 months from the first one and 4 of 6 showed a rapid disease progression [8^▪^]. Even if only some case reports are available, the transplanted patients showed on average a good survival rate with complete recovery of liver function and wellness lasting at least 6–8 months after OLT [5,24,42].

## CONCLUSION

SARS-CoV2 is a newly discovered virus and little is known about its direct cytopathic and indirect effects, including disruptive potentials on human immune system. These cholangiopathies represent an open issue in science world as their causative factors are far from being explained and understood. PBC has been related to viral infections acting as triggers, determining autoimmunity activation with consequent bile ductal lesions and cholestasis; for what concerns SSC, its pathogenesis is more uncertain and complex, leading to a severe and rapidly progressing disease.

The two cholangiopathies are sustained by different mechanisms and even if until now a few cases have been reported, they should be kept into account. Blood tests constituting AST, ALT, ALP, GGT and total bilirubin should be periodically performed during ICU stay and above all after discharge, in order to promptly detect any alteration. Other studies are necessary to understand the exact pathogenesis of these diseases and to develop new therapeutic strategies.

## Acknowledgements

*None.*

### Financial support and sponsorship

*None.*

### Conflicts of interest

*There are no conflicts of interest.*

## REFERENCES AND RECOMMENDED READING

Papers of particular interest, published within the annual period of review, have been highlighted as:

▪ of special interest
▪▪ of outstanding interest
